# Using the International Classification of Functioning, Disability and Health (ICF) to Describe Children Referred to Special Care or Paediatric Dental Services

**DOI:** 10.1371/journal.pone.0061993

**Published:** 2013-04-16

**Authors:** Denise Faulks, Johanna Norderyd, Gustavo Molina, Caoimhin Macgiolla Phadraig, Gabriela Scagnet, Caroline Eschevins, Martine Hennequin

**Affiliations:** 1 CHU Clermont-Ferrand, Service d’Odontologie, Clermont-Ferrand, France; 2 Clermont Université, Université d’Auvergne, EA3847, Centre de Recherche en Odontologie Clinique, Clermont-Ferrand, France; 3 National Oral Disability Centre, The Institute for Postgraduate Dental Education, Jönköping, Sweden; 4 CHILD, Swedish Institute for Disability Research, School of Health Sciences, Jönköping University, Jönköping, Sweden; 5 Facultad de Odontología, Universidad Nacional de Córdoba, Córdoba, Argentina; 6 Dublin Dental University Hospital, Trinity College, Dublin, Ireland; 7 Quinquela Martin Hospital, Government of Buenos Aires City & National University of Buenos Aires, Buenos Aires, Argentina; University of Toronto, Canada

## Abstract

Children in dentistry are traditionally described in terms of medical diagnosis and prevalence of oral disease. This approach gives little information regarding a child’s capacity to maintain oral health or regarding the social determinants of oral health. The biopsychosocial approach, embodied in the International Classification of Functioning, Disability and Health - Child and Youth version (ICF-CY) (WHO), provides a wider picture of a child’s real-life experience, but practical tools for the application of this model are lacking. This article describes the preliminary empirical study necessary for development of such a tool - an ICF-CY Core Set for Oral Health. An ICF-CY questionnaire was used to identify the medical, functional, social and environmental context of 218 children and adolescents referred to special care or paediatric dental services in France, Sweden, Argentina and Ireland (mean age 8 years ±3.6yrs). International Classification of Disease (ICD-10) diagnoses included disorders of the nervous system (26.1%), Down syndrome (22.0%), mental retardation (17.0%), autistic disorders (16.1%), and dental anxiety alone (11.0%). The most frequently impaired items in the ICF *Body functions* domain were ‘Intellectual functions’, ‘High-level cognitive functions’, and ‘Attention functions’. In the *Activities and Participation* domain, participation restriction was frequently reported for 25 items including ‘Handling stress’, ‘Caring for body parts’, ‘Looking after one’s health’ and ‘Speaking’. In the *Environment* domain, facilitating items included ‘Support of friends’, ‘Attitude of friends’ and ‘Support of immediate family’. One item was reported as an environmental barrier – ‘Societal attitudes’. The ICF-CY can be used to highlight common profiles of functioning, activities, participation and environment shared by children in relation to oral health, despite widely differing medical, social and geographical contexts. The results of this empirical study might be used to develop an ICF-CY Core Set for Oral Health - a holistic but practical tool for clinical and epidemiological use.

## Introduction

Poor oral health is the commonest health problem in the world and as such, is a major public health issue and a major consumer of health spending [1]. Extreme inequalities in oral health exist however, in relation to functional capacity and disability, socioeconomic status and socio-political environment, both for adults and children [2]–[10]. Studies describe poor oral health in young populations with medical, social or psychological problems but these populations are ill-defined and difficult to identify and target. Many reports describe their study population solely in terms of medical diagnosis, but this gives very little information as to the capacity of the child to maintain oral health, within his or her socio-environmental context [11]. Other studies concentrate on quantifying disease prevalence but this again gives little insight into the actual determinants of poor oral health [4]. Even the existing quality of life instruments do not accommodate the patient’s sociocultural environment [12], [13] and may not be applicable for use with certain groups with disability. In order to aid the shift towards a holistic, biopsychosocial point of view it is necessary to develop validated tools to describe a child’s functional experience, ability to participate and the environmental context in which he or she lives [14]–[17].

**Figure 1:**
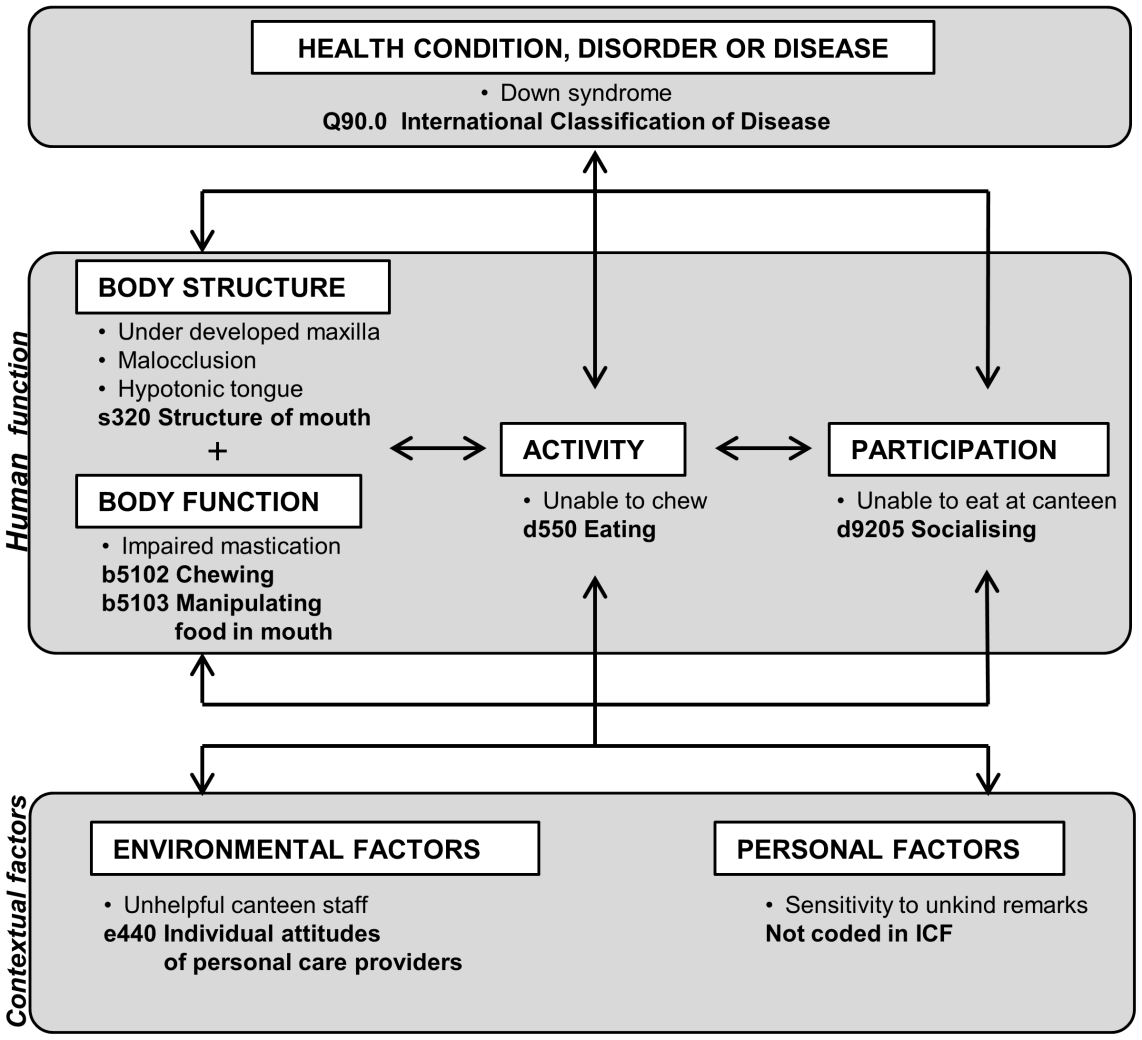
Interactions between the components of the ICF model with an oral health example.

The most comprehensive model for describing human functioning in relation to health and the environment is the International Classification of Functioning, Disability and Health (ICF), adopted by the WHO in 2001 [18] and adapted for use in children and adolescents from 2007 (Child and Youth version: ICF-CY) [19], [20]. The ICF model describes human functioning in terms of Body structure, Body function, Activities, and Participation. These aspects of human functioning influence, and are influenced by, Health condition, Environmental factors and Personal factors. The ICF model is illustrated in Figure 1, with an oral health example. The basic premise of the ICF is that it is *Universal*, i.e. is applicable to all people irrespective of health condition or cultural context. This characteristic is extremely important, as it allows comparison of equivalent health conditions of different aetiology and may reveal situational inequality or inequity in health and functioning. Further information regarding the ICF is available on the WHO website or by downloading the ICF Beginners Guide [21].

The ICF model is crystallised in the ICF classification, which provides an exhaustive list of items related to Body structure, Body function, Activities and Participation, and Environmental factors. Examples of specific items and their codes can be seen in Figure 1. Personal factors are not yet listed within the full classification. The ICF and the ICF-CY form part of the WHO Family of Classifications and are designed to be compatible with the International Classification of Disease (ICD) [22]. The ICD is an exhaustive list of human disease and the ICF was developed in part to remedy the reductive nature of such an approach. An ICF-Checklist has been developed by the WHO for standardised data collection, which consists of a list of 153 items considered to be the most relevant ICF categories for clinical purposes [23]. The ICF Checklist has been modified for use in many different domains and disciplines to collect holistic data regarding human functioning [24]–[30].

The ICF has only twice been used empirically in the domain of oral health [16], [31]. Practical use of the ICF is notoriously difficult as the classification is unwieldy and deliberately exhaustive, listing over 1400 items. The WHO solution to this problem has been to develop ICF Core Sets – reduced lists of ICF items specific to a particular domain and designed for practical use in clinical and epidemiological contexts [32]. ICF Core Sets are defined following strict methodological protocols and have been produced for over 30 different health domains so far (e.g. sleep, head and neck cancer, multiple sclerosis, obesity) [32]–[40]. One of the four requisite preliminary studies in the development of an ICF Core Set is an empirical study using a discipline-specific ICF Checklist [32]. The other preliminary studies are a systematic literature review, a qualitative study to elicit the patients’ point of view, and an expert survey exploring professional opinion.

This article describes the preliminary empirical study necessary to initiate the process of developing an ICF-CY Core Set for Oral Health. It is hoped that this process, once completed, will provide a holistic but practical tool for investigating and reporting oral health in children. The study reported here is the first to describe the functional, social and environmental profiles of an international sample of children within the specific context of oral health.

### Aims and objectives

The aims of this study were therefore:

- To undertake the preliminary empirical study necessary for the development of an ICF-CY Core Set for Oral Health.
- To describe common aspects of the medical, functional, social and environmental context of children and adolescents referred tooral health services internationally using the ICF-CY.

The objectives were:

- To collect data using the ICF-CY from a population of persons under 16 years of age referred to special care or paediatric dental services in a prospective, cross-sectional, multinational study.
- To describe the sociodemographic, medical and dental profile of this population.
- To describe the most common problems of body structure, function, activity and participation encountered by this population.
- To describe the most common environmental factors impacting on this population.

## Participants and Methods

The methodology used in this empirical study is based on that developed by the ICF Research Branch of the WHO Collaborating Centre for the Family of International Classifications (DIMDI, Germany) in partnership with the World Health Organisation Classification, Terminology and Standards group (CTS). It has been used in a large number of medical domains but never before in the field of oral health, or using the ICF-CY [24]–[30], [32].

### Ethical approval

Ethical approval for the study was given by the local authority in each of the investigation centres (France: Comité d'Ethique des Centres d'Investigation Clinique de l'Inter-région Rhône-Alpes-Auvergne; Sweden: The Regional Ethical Review Board, Linköping University; Argentina: CIEIS Comité Institucional de Etica en Investigcion en Salud, Universidad National de Cordoba; Ireland: Faculty Research Ethics Committee, Trinity College, Dublin.).

### The questionnaire

The ICF Checklist [23] was modified to give an ICF-CY Checklist for Oral Health. A point by point comparison was undertaken between ICF and ICF-CY items on the Checklist and for any differing items the ICF-CY version was adopted to accommodate for children. Other items that only exist in the ICF-CY (such as items relating to early language development or schooling) were added to the questionnaire. Finally, items specific to oral health but that did not appear in the original Checklist were added using a previous list established by Faulks & Hennequin [41]. An additional question was added to the general medical section of the questionnaire regarding perception of oral health [42], [43], as it has been proposed that oral and general health must be regarded as separate constructs [44].

The resulting ICF-CY Checklist for Oral Health (Appendix S1) recorded:

- demographic information,
- medical and dental diagnoses using the International Classification of Diseases (ICD) [22] and the International Classification of Diseases – Application to Dentistry and Stomatology (ICD-DA) [45],
- information regarding other health related issues (use of medication, need for assistance in daily living),
- patient and/or carer subjective perception of physical, mental and oral health,
- presence or absence of an impairment for a list of items from the *Body Functions* component of the ICF-CY (43 items),
- presence or absence of an impairment for a list of items from the *Body Structures* component of the ICF-CY (23 items),
- presence or absence of restriction in participation for a list of items from the *Activities and Participation* component of the ICF-CY (37 items),
- presence of a barrier or of a facilitating factor (facilitator) for a list of items from the *Environmental* component of the ICF-CY (25 items),
- other relevant contextual information completed free-hand by the investigator.

Items of the ICF-CY were evaluated according to age-related expectations within the child’s cultural context and current environment, thus some items were not applied for younger children. The parents or carers were invited to compare the child’s activity with that of a sibling at the same age, as family culture may dictate at what age a child is expected to clean his or her teeth independently, for example. National differences were also evoked by the investigators, particularly in the accepted age for a child to go to a local shop (‘Acquisition of goods and services’) or to help out with the preparation of meals. The integration of cultural differences into the ICF model is a founding principle of the classification, as the child can only be considered restricted in participation for those activities that are expected of him/her in his or her sociocultural role.

The ICF-CY Checklist for Oral Health was produced in English, French, Spanish and Swedish, using pre-existing WHO translations of ICF items.

### Training of the investigators

The investigators were brought together for a training programme on the use of the ICF and on the use of the ICF-CY Checklist for Oral Health in particular. Training was given using case studies, item by item examples, and peer review of questionnaire completion to ensure consensus and consistency. Inter-rater reliability was not formally tested however. Calibration of the investigators involving repeat examination and interview of the same children was not feasible in terms of time constraints, or in terms of the difficulty organising a clinical session for an international group of investigators. It is also likely that ethical considerations would have restricted participation of vulnerable children in such a calibration exercise.

### The study population

This study used the ICF-CY Checklist for Oral Health to describe the medical, functional, social and environmental context of children and adolescents referred to special care or paediatric dental services. This population was chosen for data collection, as it was assumed that these children accumulate a higher prevalence of potential risk factors for poor oral health than the general child population. Children are generally referred to services because they are dentally fearful, because of a disability that directly or indirectly affects their oral health [41], or because of the magnitude of treatment required, either in terms of quantity or severity of oral pathology [46].

Under the assumption of an equal effects model (EEM) [47], a power (1-β) of 0,8 and a level of significance (α) of 0,05 a sample size of 194 individuals was necessary in order to determine frequencies of ICF items with a precision of 10%. The aim was therefore to recruit a convenience sample of 200 children or adolescents referred to Paediatric or Special Care Dental Units in France, Sweden, Ireland and Argentina.

The inclusion criteria were:

i) Patient under 16 years of age on the day of data collection.

ii) Patient referred to a Paediatric or Special Care Dental Unit.

iii) Patient with a signed consent form. Informed consent was sought from patients and/or their legal representatives for anonymous data collection and analysis.

### Data collection

All patients fulfilling the inclusion criteria were recruited consecutively, in order of presentation to the service on the days when the investigator was present, except in Ireland where a ‘gatekeeper’ system was imposed by local ethical committee regulations. In this centre potential participants had to be informed of the study by a gatekeeper and agree to be approached prior to contact from the investigator. The ICF-CY Checklist for Oral Health was completed by the investigator based on a structured interview with the patient and/or primary carer, from direct observation of the patient, and from information in the medical/dental records. In case of discrepancy between carer report and direct observation of the child’s ability, the investigator used clinical judgement as to the degree of impairment or activity limitation. In case of doubt, discussion between investigators was used to reach consensus. The ICF-CY Checklist for Oral Health took approximately 30 minutes to complete for each patient.

### Data entry and analysis

Central, double, data entry was performed using Microsoft Excel®. Descriptive statistics were used to describe the study population and to examine the frequency of problems recorded by the ICF-CY Checklist for Oral Health using SPSS®. For the ICF-CY components *Body Functions*, *Body Structures* and *Activities and Participation* absolute frequencies and relative frequencies (prevalence) of impairment/limitation in the study population were calculated. For *Environmental Factors*, absolute frequencies and relative frequencies (prevalence) of items entered as either a barrier or facilitator were reported. The frequency with which an item was reported for each country was compared using a χ^2^ test.

## Results

### Study population

218 patients were included in the study, of which 37.6% (82) were recruited in France, 25.7% (56) in Sweden, 25.3% (55) in Argentina and 11.5% (25) in Ireland.

The ICF-CY Checklist for Oral Health was completed by the investigator with help from the parent(s) in 97.2% (212) cases, using direct observation of the child in 90.4% (197) cases and using the medical and dental notes in 84.9% (185) cases. Only 18 patients (8.3%) were able to reply to the questions for themselves.

Demographic and general medical and functional information is presented in Table 1. Significant differences were found between countries for certain daytime activities and for items relating to general medical and functional management (χ^2^ test).

**Table 1:** Demographic, general medical and functional information.

	INTERNATIONAL	FRANCE	SWEDEN	ARGENTINA	IRELAND	
**Number of participants**	**n = 218**	n = 82	n = 56	n = 55	n = 25	?^2^ test
**Mean age (±SD)**	**8.7 yrs (±3.58yrs)**	8.7 yrs (±3.34yrs)	8.6 yrs (±4.09yrs)	8.5 yrs (±3.44yrs)	9.0 yrs (±3.64yrs)	ns (Anova)
**Age range**	**4 mths to 15 yrs 11 mths**	15 mths to 15 yrs 11 mths	22 mths to 15 yrs 6 mths	4 mths to 15 yrs 9 mths	31 mths to 15 yrs 11 mths	
**Female sex**	**34.4%**	35.4%	33.9%	30.9%	40.0%	ns
**Daytime activity/schooling**						
Home	**6.4%**	3.7%	7.1%	12.7%	0%	na
Preschool childcare	**10.6%**	1.2%	25.0%	12.7%	4.0%	na
Mainstream schooling	**29.8%**	42.7%	12.5%	18.2%	52.0%	p<0.001
Special schooling	**43.1%**	36.6%	51.8%	54.5%	20.0%	p<0.05
Other	**10.1%**	15.8%	3.6%	1.8%	24.0%	na
**Significant medical diagnosis (ICD 10)**	**97.7%**	93.9%	100%	100%	100%	na
Anxiety/phobia unique ICD 10 diagnosis	**11.0%**	24.4%	0%	0%	16.0%	na
Problem at birth	**41.7%**	31.7%	64.3%	30.9%	48.0%	p<0.001
Regular medication	**47.7%**	39.0%	66.1%	45.4%	40.0%	p<0.01
Assistive devices	**59.2%**	54.9%	94.6%	30.9%	56.0%	p<0.001
Assistance for daily living	**74.3%**	68.3%	92.9%	80.0%	40.0%	p<0.001
Paramedical treatment (speech therapy, physiotherapy…)	**66.1%**	70.7%	41.1%	89.1%	56.0%	p<0.001
**Significant dental diagnosis (ICD-DA)**	**90.8%**	86.6%	83.9%	100%	100%	na

χ^2^ test: statistical difference between countries; ns  =  non-significant; na  =  non-applicable.

The perceived quality of physical, mental and oral health of the patients is shown in Table 2. Reported rates of moderate, poor and very poor health were significantly different between countries for physical, mental and oral health (χ^2^ test).

**Table 2:** Subjective perception of physical, mental and oral health.

	INTERNATIONAL	FRANCE	SWEDEN	ARGENTINA	IRELAND	
Number of participants	n = 218	n = 82	n = 56	n = 55	n = 25	?^2^ test
**Physical health**						
Very Good	**33.5%**	36.6%	33.9%	20%	52.0%	
Good	**45.9%**	56.1%	30.4%	54.5%	28.0%	
Moderate	**17.4%**	7.3%	26.8%	23.6%	16.0%	
Poor	**2.3%**	0%	5.4%	1.8%	4.0%	
Very Poor	**0.9%**	0%	3.6%	0%	0%	
Moderate+Poor+Very Poor	**20.6%**	7.3%	35.7%	25.5%	20.0%	p<0.001
**Mental Health**						
Very Good	**31.7%**	45.1%	30.4%	7.2%	44.0%	
Good	**37.2%**	34.1%	39.3%	45.5%	24.0%	
Moderate	**27.1%**	19.5%	23.2%	41.8%	28.0%	
Poor	**3.7%**	1.2%	5.4%	5.4%	4.0%	
Very Poor	**0.5%**	0%	1.8%	0%	0%	
Moderate+Poor+Very Poor	**31.2%**	20.7%	30.4%	47.3%	32.0%	p<0.05
**Oral Health**						
Excellent	**9.2%**	4.9%	19.6%	0%	20%	
Very Good	**20.2%**	17.1%	33.9%	14.5%	12.0%	
Good	**28.4%**	34.1%	26.8%	27.2%	16.0%	
Moderate	**20.6%**	26.8%	17.9%	20%	8.0%	
Poor	**14.7%**	8.5%	1.8%	32.7%	24.0%	
Very Poor	**6.9%**	8.5%	0%	5.4%	20.0%	
Moderate+Poor+Very Poor	**42.2%**	43.9%	19.6%	58.2%	52.0%	p<0.001

χ^2^ test: statistical difference between countries.

The 213 patients (97.7%) with at least one ICD-10 medical diagnosis are described in Table 3. The most common diagnosis was chromosomal abnormalities (31.2%) of which 22.0% was accounted for by Down syndrome. Other common diagnoses included disorders of the nervous system (26.1%), mental retardation (17.0%), disorders of psychological development including autism (16.1%) and episodic or paroxysmal disorders (14.2%). 11.0% of patients presented dental anxiety or phobia without other health conditions. The prevalence of the main ICD domains reported differed significantly between countries (χ^2^ test).

**Table 3:** Description of the study population according to ICD-10 diagnosis (multiple diagnoses per patient possible).

ICD DOMAIN or ICD sub-domain	International	France	Sweden	Argentina	Ireland	
Number of participants	n = 218	n = 82	n = 56	n = 55	n = 25	?2 test
**MENTAL AND BEHAVIOURAL DISORDERS**	**50.9% (111)**	51.2% (42)	67.9% (38)	30.9% (17)	56.0% (14)	p<0.01
Neurotic, stress-related and somatoform disorders	**13.3% (29)**	13.3% (24)	1.8% (1)	0%	16.0% (4)	na
Dental anxiety or phobia unique diagnosis	**11.0% (24)**	24.4% (20)	0%	0%	16.0% (4)	na
Mental retardation	**17.0% (37)**	4.8% (4)	42.9% (24)	7.2% (4)	20.0% (5)	na
Disorders of psychological development (autism etc.)	**16.1% (35)**	12.2% (10)	14.3% (8)	21.8% (12)	20.0% (5)	na
**CONGENITAL MALFORMATIONS, DEFORMATIONS AND CHROMOSOMAL ABNORMALITIES**	**59.2% (129)**	52.4% (43)	82.1% (46)	50.9% (28)	48% (12)	p<0.001
Congenital malformations of the nervous system	**10.1% (22)**	6.1% (5)	16.1% (9)	12.7% (7)	4.0% (1)	na
Congenital malformations of the circulatory system	**10.6% (23)**	10.9% (9)	17.9% (10)	0%	16.0% (4)	na
All chromosomal abnormalities including Down syndrome	**31.2% (68)**	34.1% (28)	26.8% (15)	32.7% (18)	28.0% (7)	ns
Down syndrome	**22.0% (48)**	18.3% (15)	23.2% (13)	27.3% (15)	20.0% (5)	ns
**DISEASES OF THE NERVOUS SYSTEM**	**26.1% (57)**	12.1% (10)	44.6% (25)	34.5% (19)	12.0% (3)	p<0.001
Episodic and paroxysmal disorders	**14.2% (31)**	10.9% (9)	23.2% (13)	12.7% (7)	8.0% (2)	na
Cerebral palsy and paralytic syndromes	**8.7% (19)**	0%	12.5% (7)	20.0% (11)	4.0% (1)	na
**ENDOCRINE, NUTRITIONAL AND METABOLIC DISORDERS**	**5.5% (12)**	8.5% (7)	3,6% (2)	3.6% (2)	4.0% (1)	na
OTHER	**20.2% (44)**	14.6% (12)	19.6% (11)	12.7% (7)	17.4% (14)	p<0.001
NONE	**2.3% (5)**	6.1% (5)	0%	0%	0%	na

χ^2^ test: statistical difference between countries; ns  =  non-significant; na  =  non-applicable.

198 patients (90.8%) had an ICD-DA dental diagnosis of which the most common diagnoses were dentofacial anomaly or malocclusion (44.0%) and dental caries (42.6%) (Table 4). The prevalence of these disorders differed significantly between countries (χ^2^ test).

**Table 4:** Description of the patients according to ICD-DA diagnosis (multiple diagnoses per patient possible).

ICD-DA sub-domain	INTERNATIONAL	FRANCE	SWEDEN	ARGENTINA	IRELAND	
Number of participants	**n = 218**	n = 82	n = 56	n = 55	n = 25	?^2^ test
Dental caries	**42.6% (93)**	42.7% (35)	10.7% (6)	69.1% (38)	56.0% (14)	p<0.001
Dentofacial anomalies including malocclusion	**44.0% (96)**	36.5% (30)	62.5% (35)	49.1% (27)	16.0% (4)	p<0.001
None	**9.2% (20)**	13.4% (11)	16.1% (9)	0%	0%	na

χ^2^ test: statistical difference between countries; ns  =  non-significant; na  =  non-applicable.

### ICF-CY Items

Overall, 114 of the 128 categories in the ICF-CY Checklist for Oral Health were identified as at least mildly impaired in 10% or more of the study population (89.1%). A total of 56 categories were identified as at least mildly impaired in over 50% of the study population (40.6% of items).

The most frequently impaired were ‘Intellectual functions’, ‘High-level cognitive functions’, ‘Attention functions’ and ‘Mental functions of language’ (cited by over 70% of the international population) (Table 5). Table 5 also shows the frequency of impairment reported for items of oral function. Oral function was impaired in a substantial minority of the international population, particularly ‘Manipulation of food in the mouth’ (43%), ‘Chewing function’ (40%) and ‘Biting function’ (39%).

**Table 5:** Frequency of impairment in the ICF “Body functions” component.

		INTERNATIONAL	FRANCE	SWEDEN	ARGENTINA	IRELAND	
Number of participants		n = 218	n = 82	n = 56	n = 55	n = 25	?^2^ test
**ICF code**	**Items cited by over 50% of patients in the international study (n = 10)**						
b117	Intellectual functions	**76%**	67%	89%	89%	48%	p<0.001
b164	High-level cognitive functions	**74%**	68%	86%	87%	42%	p<0.001
b140	Attention functions	**72%**	67%	75%	80%	60%	ns
b167	Mental functions of language	**71%**	63%	77%	82%	56%	p<0.05
b152	Emotional functions	**65%**	68%	54%	84%	*36%*	p<0.001
b114	Orientation functions	**62%**	59%	73%	76%	*32%*	p<0.01
b147	Psychomotor functions	**62%**	61%	*46%*	85%	52%	p<0.001
b122	Global psychosocial functions	**59%**	*48%*	*61%*	73%	60%	p<0.05
b144	Memory functions	**54%**	*44%*	*43%*	85%	*40%*	p<0.001
b130	Energy and drive functions	**52%**	*45%*	*73%*	53%	*36%*	p<0.05
**ICF code**	**Items relating to oral function**						
b250	Taste function	***19%***	*20%*	*14%*	*26%*	*12%*	na
b5100	Sucking function	***23%***	*9%*	*32%*	*35%*	*20%*	p<0.001
b5101	Biting function (front teeth)	***39%***	*39%*	*41%*	*47%*	*16%*	ns
b5102	Chewing function (back teeth)	***40%***	*43%*	*30%*	*47%*	*32%*	ns
b5103	Manipulation of food in mouth	***43%***	*39%*	*46%*	53%	*28%*	ns
b5105	Swallow function	***28%***	*17%*	*36%*	*38%*	*24%*	p<0.05

*Italics if<50%*; χ2 test: statistical difference between countries; ns  =  non-significant; na  =  non-applicable

In the Body structures component only one item was impaired in over 50% of patients - ‘Structure of the teeth’ (Table 6).

**Table 6:** Frequency of impairment in the ICF “Body structures” component.

		INTERNATIONAL	FRANCE	SWEDEN	ARGENTINA	IRELAND	
Number of participants		n = 218	n = 82	n = 56	n = 55	n = 25	?^2^ test
**ICF code**	**Items cited by over 50% of patients in the international study (n = 1)**						
s3200	Structure of the teeth	**58%**	70%	*39%*	65%	*40%*	p<0.001

*Italics if<50%*; χ^2^ test: statistical difference between countries; ns  =  non-significant; na  =  non-applicable.

In the Activities and Participation component, participation restriction in the patient’s current environment is shown in Table 7. Eight items were cited by 70% of patients or more in the international population (of those for whom the item was age-relevant): ‘Preparing meals’, ‘Undertaking multiple tasks’, ‘Acquiring skills’, ‘Solving problems’, ‘Handling stress’, ‘Caring for body parts’, ‘Looking after one’s health’ and ‘Speaking’.

**Table 7:** Frequency of participation restriction, in current environment and in relation to age, in the ICF “Activities and participation” component.

		INTERNATIONAL		FRANCE		SWEDEN		ARGENTINA		IRELAND		
Items cited by over 50% of patients in the international study (n = 25 items)		n replies	% restricted	n replies	% restricted	n replies	% restricted	n replies	% restricted	n replies	% restricted	?^2^ test
d630	Preparing meals	**46**	**74%**	15	73%	15	67%	10	100%	6	50%	na
d220	Undertaking multiple tasks	**216**	**73%**	81	68%	56	75%	55	87%	24	54%	p<0.05
d155	Acquiring skills	**217**	**72%**	81	63%	56	80%	55	85%	25	56%	p<0.01
d175	Solving problems	**217**	**72%**	81	65%	56	75%	55	87%	25	60%	p<0.05
d240	Handling stress/psychological demands	**216**	**72%**	81	82%	56	55%	54	87%	25	52%	p<0.001
d520	Caring for body parts	**215**	**73%**	81	68%	56	79%	54	87%	24	50%	p<0.01
d570	Looking after one’s health	**177**	**72%**	79	67%	19	68%	54	93%	25	*28%*	p<0.001
d330	Speaking	**216**	**71%**	81	68%	56	79%	54	83%	25	*36%*	p<0.001
d620	Acquisition of goods and services	**154**	**69%**	78	67%	19	79%	35	86%	22	50%	p<0.05
d177	Making decisions	**217**	**68%**	81	63%	56	70%	55	85%	25	*44%*	p<0.01
d230	Carrying out daily routine	**216**	**67%**	81	57%	56	73%	54	87%	25	*44%*	p<0.001
d250	Managing one’s own behaviour	**216**	**65%**	81	82%	56	54%	54	83%	25	*44%*	p<0.001
d510	Washing oneself	**215**	**65%**	81	59%	56	75%	54	74%	24	*42%*	p<0.01
d720	Complex interpersonal interactions	**216**	**64%**	81	62%	56	54%	54	85%	25	52%	p<0.01
d9	Community, social and civic life	**210**	**63%**	81	58%	50	70%	54	80%	25	*40%*	p<0.01
d210	Undertaking a single task	**217**	**62%**	81	64%	56	57%	55	76%	25	*44%*	p<0.01
d710	Basic interpersonal interactions	**218**	**62%**	82	61%	56	50%	55	80%	25	56%	p<0.01
d310	Communicating with spoken messages	**218**	**58%**	82	59%	56	54%	55	71%	25	*40%*	ns
d730	Relating with strangers	**217**	**57%**	81	55%	56	*25%*	55	87%	25	*48%*	p<0.001
d331	Pre-talking, babbling	**11**	**55%**	5	40%	2	100%	3	67%	1	*0%*	na
d335	Producing nonverbal messages	**218**	**55%**	82	62%	56	*41%*	55	69%	25	*28%*	p<0.001
d740	Formal relationships	**211**	**55%**	80	66%	54	*24%*	54	81%	23	*39%*	p<0.001
d315	Communicating with nonverbal messages	**218**	**51%**	82	51%	56	*39%*	55	76%	25	*24%*	p<0.001
d820	School education	**198**	**54%**	80	*36%*	39	*44%*	54	83%	25	60%	p<0.001
d440	Fine hand use	**217**	**50%**	82	55%	56	63%	54	*46%*	25	*36%*	ns

χ^2^ test: statistical difference between countries; ns  =  non-significant; na  =  non-applicable; *Italics if<50%*.

In the Environment component, 20 items were reported as having an impact as either a facilitator or a barrier for over 50% of patients (Table 8). The three most frequently cited facilitating items were ‘Support of friends’, ‘Attitude of friends’ and ‘Support of immediate family’. Only one item was reported in over 50% of the international population as an environmental barrier – ‘Societal attitudes’, although there was a significant difference between countries for this item (p<0.001).

**Table 8:** Items of the “Environment” component with an impact for >50% of patients.

		INTERNATIONAL		FRANCE		SWEDEN		ARGENTINA		IRELAND		
Items cited by over 50% of patients in the international study (n = 20 items)		% impact	% facilitation	% impact	% facilitation	% impact	% facilitation	% impact	% facilitation	% impact	% facilitation	?^2^ test
e320	Support of friends	**90%**	**81%**	95%	90%	91%	76%	84%	67%	84%	76%	na
e420	Attitude of friends	**90%**	**86%**	94%	91%	95%	92%	84%	72%	80%	85%	na
e310	Support of immediate family	**89%**	**93%**	93%	88%	100%	100%	71%	92%	96%	92%	na
e355	Support of health professionals	**88%**	**94%**	95%	87%	98%	100%	76%	100%	68%	88%	na
e410	Attitude of immediate family	**88%**	**88%**	95%	83%	98%	96%	67%	81%	84%	95%	na
e450	Attitude of health professionals	**88%**	**88%**	94%	78%	96%	100%	78%	91%	68%	88%	na
e570	Social security services, systems and policies	**87%**	**66%**	92%	88%	86%	85%	89%	*14%*	72%	67%	na
e580	Health services, systems and policies	**87%**	**67%**	94%	67%	96%	98%	76%	*29%*	64%	63%	na
e330	Support of people in position of authority	**86%**	**81%**	90%	84%	96%	70%	67%	89%	92%	83%	na
e340	Support of personal care providers and assistants	**79%**	**96%**	79%	97%	80%	98%	75%	98%	84%	86%	ns
e440	Attitude of personal care providers and assistants	**78%**	**91%**	81%	89%	79%	95%	78%	88%	68%	94%	ns
e455	Attitude of health-related professionals	**75%**	**88%**	83%	88%	59%	88%	80%	89%	72%	89%	p<0.05
e360	Support of other professionals	**73%**	**85%**	84%	91%	54%	90%	73%	85%	76%	53%	p<0.01
e465	Social norms, practices and ideologies	**71%**	**59%**	*46%*	*42%*	88%	80%	89%	63%	72%	*28%*	p<0.001
e460*	Societal attitudes*	**67%**	***43%***	54%	*9%*	71%	55%	93%	57%	56%	57%	p<0.001
e575	General social support services, systems and policies	**66%**	**62%**	55%	84%	70%	87%	84%	*22%*	52%	*46%*	p<0.01
e586	Special education and training services, systems and policies	**61%**	**75%**	51%	79%	*43%*	75*%*	93%	71%	64%	81%	p<0.001
e540	Transportation services, systems and policies	**59%**	**65%**	*48%*	87%	64%	92%	78%	*19%*	44%	82%	p<0.01
e1101	Drugs	**53%**	**94%**	*40%*	100%	63%	83%	51%	96%	56%	100%	p<0.05
e115	Products and technology for personal use in daily living	**51%**	**98%**	*49%*	100%	63%	97%	55%	97%	40%	100%	ns

% impact  =  overall impact of the factor; % facilitation  =  positive impact of the factor; χ^2^ test: statistical difference between countries; ns  =  non-significant; na  =  non-applicable; Italics if<50%.* Only one item (e460) was more often reported as a barrier than a facilitator.

## Discussion

This prospective, international study describes a population of 218 children and adolescents referred to special care or paediatric dental services. The ICF-CY was used to identify aspects that are common to children and adolescents attending oral health services with different health conditions and in different socio-cultural contexts. Overall, the results show that this population has common functional, social and environmental profiles, with 56 ICF-CY categories being identified in over 50% of the study population. The fact that these categories were cited so often, in such a heterogeneous population, confirms that certain items of functional impairment and participation restriction are particularly relevant to oral health. The results of this empirical study need now to be confronted with those from other preliminary studies before consensus on an ICF-CY Core Set in Oral Health can be reached [32].

The study provides a detailed description of children referred to secondary oral health services. A significant medical diagnosis, over and above anxiety, was reported for 86.7% of the study population. However, medical diagnosis alone is insufficient to quantify or qualify the degree to which the maintenance of oral health and the receipt of dental care might be difficult. In particular, the danger of extrapolating medical diagnoses to reflect individual patient experience is illustrated by the fact that 79.4% and 68.8% of the population were perceived by parents as having good or very good physical health and mental health respectively. Positive reporting of health and quality of life within populations with disability has been referred to as the ‘disability paradox’ and is a common finding [48]. This finding is also likely to reflect the fact that professionals tend to look for impairments in a child’s functioning, whereas parents and carers will look at the positive aspects of a child’s participation, seeing ‘beyond’ the medical diagnosis.

In terms of oral health, disease prevalence was high with 42.6% of the population presenting dental caries (treated and untreated) and 44.0% presenting dentofacial anomalies including malocclusion, as defined by the ICD. It is widely recognised that oral health is generally poorer in children with special health needs than in the general population [8], [49]. In addition, all the children included in the study were attending services and therefore had a perceived need, even in Sweden where patients were referred and recalled for preventive care due to being considered ‘at risk’ by the paediatric and dental teams. No direct comparison can be made between the caries prevalence reported here and caries rates reported for the general population of the study countries because the study populations are very different. However, in Sweden, where the caries rate was lowest, early intensive preventive intervention seems to have a positive effect for children with disability. It must be remembered, however, that the Swedish population reported here did not include any children with dental anxiety only, or any children referred to secondary services for extensive treatment under general anaesthesia, so the caries rates could be expected to be different to those of the other study centres. These results are interesting as they suggest that children with significant medical diagnoses may be excluded from mainstream oral health preventive measures in many countries, but that positive intervention might be used to address these underlying situations of inequality.

Despite high prevalence of oral disorders, 57.8% of patients in the current study were perceived by themselves or their parents as having excellent, very good or good oral health. This may be compared to a previous investigation of perception of oral health, where 65.6% of children presenting with dental caries and 86.7% of children presenting with cleft lip and/or palate but with no other health conditions, reported good oral health [50]. The literature confirms the validity of such single-item proxy measures [43], [51]–[53] and also the tendency for persons to maintain a positive sense of well-being when coping with oral disability [17]. It is also in line with the WHO’s definition of health where oral health is described as a state of physical, psychological and social well-being, not merely the absence of disease [54].

All the frequently cited items in the ICF ‘Body Functions’ domain were derived from the ‘Mental Functions’ chapter, with particularly high frequency for items related to intellectual and cognitive function, attention, mental functions of language and emotion. Thus maintenance of oral health seems more related to the cognitive ability to comprehend daily oral care and cope with examination and treatment than to specific medical diagnoses. This corresponds with previous studies of the ICF profile of disabled patients with difficulty tolerating dental treatment or patients requiring treatment under general anaesthesia [16], [31]. Oral function was not identified in the top ten impairments in the current study despite the fact that a high level of oral impairment was reported with 40% of children reporting impaired chewing and 28% impaired swallowing. Prevalence rates for problems with swallowing and digesting food for children with special health care needs have previously been cited at 28% to 8% [55], but it is not surprising that the rates found here are higher given that all were attending oral health services. 58% of patients reported impaired structure of the teeth, and this can be assumed to be linked to the high level of patients presenting dental caries (43%) and/or diseases of dental hard tissue, such as enamel defects. It is interesting that no other, more physical domains of function were cited frequently, such as movement function which could be anticipated to affect ability to maintain oral hygiene. The lack of a link between manual dexterity and oral hygiene was noted by Martens et al. [56], who hypothesised that the most agile children received less help with brushing and that this might explain higher plaque scores in this group.

The items most frequently cited in the Activities and Participation domain were distributed amongst all 9 chapters of the domain. Restricted participation was related to the ‘General tasks and demands’ chapter, such as, ‘Undertaking multiple tasks’, ‘Handling stress and psychological demands’ and ‘Managing one’s own behaviour’. It is important to note that restriction in these activities is likely to be demonstrated not only by children with a recognised medical condition, but also by those exhibiting anxiety. The ‘Communication’ chapter was also frequently cited, with 71% of the children being restricted in participation by difficulties speaking. This was reflected in the children’s reduced ability to appropriately manage ‘Interpersonal interactions and relationships’. On a more practical side, the children were limited in activities related to self-care, such as washing themselves and cleaning their teeth. This echoes results of a previous study, where young persons with Down syndrome were found to have more difficulty in performing all acts of hygiene and health care than their siblings [57].

It was enlightening to find that all except one item highlighted in the Environmental Factors domain were rated as facilitators (positive environmental factors as opposed to barriers). These facilitators were firmly embedded in the social context of the child – the support and attitudes of friends, family and health professionals. The vast majority of patients and their parents also acknowledged the help received from various services, systems and policies within their national context, despite these services varying greatly between the different countries in the study. These results are comparable to those found in a similar, multicentre study using a modified ICF Checklist to investigate patients with head and neck cancer [26]. The one barrier, cited in 57% of cases, was that of societal attitude. This item was cited as a barrier by 62% of patients with head and neck cancer, and was again one of the rare barriers reported [26]. Ableism is defined as overt discrimination against people with disability and unfortunately, this problem seemed to be an important feature of the children’s environmental and social context [58]. Discrimination and exclusion are recognised as powerful social determinants of health [59].

When comparing the results of different countries, the differences demonstrated in prevalence of items were to be expected given that all countries have different health system structures. These differences included pathways for referral to secondary care; provision of special care or paediatric care for children with disabilities; financial reimbursement of patients; active recall of patients; structural setting (hospital/clinic/community/university); financial, human and structural resources; structured targeting of certain populations; integration of oral and general medical health services; and different national levels of oral disease. In addition, existing services in some countries are demand led and therefore the profile of patients presenting to services may be different even between regions or neighbouring services. The inter-country differences may, however, reflect the fact that children with an equal level of impairment may be more or less disadvantaged, depending on their social and environmental context. Another potential reason for inter-country differences was inter-investigator variability. A previous study has demonstrated high reliability between investigators when assigning ICF codes to children with special health care needs from parental report using structured interview [60]. However, in the current study, inter-rater reliability was not controlled, although all investigators participated together in a case-based ICF training session prior to data collection.

This study is limited in its scope by design as a convenience sample was used. The study population was consciously limited to those referred to secondary services, as it was assumed that these children accumulate a higher prevalence of potential risk factors for poor oral health than the general population. However, this meant that other groups not attending services, or able to attend mainstream services, were missed. For example, it is recognised that children from socially deprived backgrounds are often poor dental attenders [61], although this might have been compensated for by the fact that in two of the study centres such children were referred directly to the unit from social services or school screening programmes in anticipation of treatment under general anaesthesia. It should be noted that this study was not designed to argue the need for specialist care but simply to describe the contextual factors affecting a group of children with high potential oral health needs. Another limiting characteristic of the current study was that data collection with regards to environmental context was restricted to those items listed in the ICF Checklist. This limits extrapolation of results in terms of the social determinants of health, as an important variable is socioeconomic status which was not measured [62].

### Perspectives

The ICF-CY Checklist for Oral Health used here is one of the first ICF-CY based tools to be developed in any medical domain. The potential for practical application of the ICF in child populations has been regularly evoked [19], [63]–[67] but rarely put into action [60], [68], [69]. The ICF-CY Checklist for Oral Health is the only questionnaire to date in the domain of oral health designed to give a holistic, biopsychosocial description of an individual, encompassing medical, functional, social and environmental context. This tool was used to collect data in different clinical contexts, in different countries and in different languages, demonstrating that the ICF is adapted for use internationally. In addition, the ICF-CY was applicable for children with a huge range of different types of impairments and ICD-10 diagnoses, confirming the robust, universal nature of the ICF items over a wide spectrum of human functioning. The universal coherence of the ICF model was confirmed, as the results describe similar profiles relating to health conditions of different aetiology, and suggest different levels of disadvantage in different national contexts. The ICF-CY Checklist for Oral Health therefore proved feasible for use in a research context, but it remains a time-consuming and unwieldy tool.

The current study may serve as a first step in the formal development of an ICF-CY Core Set for Oral Health, helping to guide the consensus process of retaining the most relevant items of the ICF-CY to oral health. A resulting Core Set would provide a practical tool for holistic assessment, as has been the case in other health domains [32]. An ICF-CY Core Set in Oral Health could be used to identify children requiring support to maintain their oral health either at the individual or the population level, encouraging targeted intervention in countries where the health care system permits. Medical and paramedical professionals could use the tool to actively screen for oral health problems in children presenting with functional and/or social problems [70]. This would help prevent poor oral health becoming an additional disability for certain disadvantaged children, a well described problem in the adult population [71]. The ICF-CY Core Set in Oral Health might also be used as an outcome measure of the impact on oral health of dental and of general services, systems and policies. In addition, the use of the universal ICF model in the assessment of children may help to improve awareness of the wider determinants of health and encourage the search for universal solutions in terms of prevention and in terms of treatment for this population. Improved description of children and adolescents requiring special care may also facilitate clinical research, which in turn may inform service provision and be used within public health and social policy arenas to address inequalities within the child population.

## Conclusion

This study demonstrates that the ICF, universal approach to health can be used to identify common profiles of functioning, activities, participation and environment encountered within the specific context of oral health, by children with a very wide range of medical diagnoses and socio-cultural contexts. This empirical study represents the first stage in the development of an ICF-CY Core Set in Oral Health. It is hoped that a Core Set will serve as a practical tool to give insight into the wider determinants of oral health and be used within public health and social policy arenas to help address inequalities within the child population.

## Supporting Information

Appendix S1 List of items contained within the ICF-CY Checklist for Oral Health.
(DOC)
