# Supplementary material for: Using the International Classification of Functioning, Disability and Health (ICF) to Describe Children Referred to Special Care or Paediatric Dental Services
Source: PLoS One. 2013 Apr 16;8(4):e61993. doi: 10.1371/journal.pone.0061993 (PMC3628581; doi:10.1371/journal.pone.0061993)
Supplement: Appendix S1 — List of items contained within the ICF-CY Checklist for Oral Health. (DOC) [file pone.0061993.s001.doc]

**LIST OF ITEMS CONTAINED WITHIN THE ICF-CY CHECKLIST FOR ORAL HEALTH**

**Demographic Information**

**Data Source** Written records / Primary respondent / Parent or guardian / Other informant / Direct observation

**Sex**

**Date of Birth**

**Residence** Home / Institution / Hospital

**Current education** Preschool child care / Mainstream schooling / Home / Special schooling / Other

**Medical diagnosis** List medical conditions and ICD codes

**Dental diagnosis** List dental conditions and ICD-DA codes

**BRIEF HEALTH INFORMATION**

**Height & weight**

How would you rate the child’s **physical health** in the past month?

How would you rate the child’s **mental and emotional health** in the past month?

How would you rate the child’s overall **oral health?**

Does the child currently have any **disease(s) or disorder(s)?**

Were there any **problems at birth**?

Has the child ever been **hospitalised**?

Is the child taking any **medication?**

Does the child have any **significant injuries?**

Does the child use any **assistive devices** such as glasses, hearing aid, wheelchair, communication board etc.?

Does **the child have assistance from anybody** with self-care or daily activities?

Is the child receiving any kind of **treatment for his or her health?**

Additional significant information on the child’s **past or present health?**

In the past month has the child **cut back** on his or her **usual activities** because of his or her health condition?

In the past month has the child been **totally unable to carry out his or her usual activities** because of his or her health condition?

**ICF-CY ITEMS**

**body Structures**

**s3200** Teeth

**s3201** Gums

**s3202** Palate

**s3203** Tongue

**s3204** Lip

**s330** Structure of the pharynx

**s510** Structure of salivary glands

**s110** Brain

**s1106** Cranial nerves

**s120** Spinal cord and peripheral nerves

**s2** Eye, ear and related structures

**s310** Nose

**s340** Larynx

**s410** Cardiovascular system

**s430** Respiratory system

**s520** Oesophagus

**s710** Head and neck region

**s720** Shoulder region

**s730** Upper extremity

**s740** Pelvic region

**s750** Lower extremity

**s760** Trunk

**s8** Structures of skin

Other body structures

**BODY FUNCTIONS**

**b250** Taste function

**b5100** Sucking

**b5101** Biting (front teeth)

**b5102** Chewing (back teeth)

**b5103** Manipulation of food in the mouth

**b5104** Salivation

**b5105** Swallowing

**b110** Consciousness functions

**b114** Orientation functions

**b117** Intellectual functions

**b122** Global psychosocial functions

**b130** Energy and drive functions

**b140** Attention functions

**b144** Memory functions

**b147** Psychomotor functions

**b152** Emotional functions

**b156** Perceptual functions

**b164** Higher level cognitive functions

**b167** Mental functions of language

**b210** Seeing functions

**b230** Hearing functions

**b255** Smelling functions

**b260** Proprioceptive functions

**b265** Touch functions

**b270** Sensory functions

**b280** Sensation of pain

**b310** Voice functions

**b410** Heart functions

**b430** Haematological functions

**b435** Immunological functions

**b440** Respiration functions

**b450** Additional respiratory functions

**b515** Digestive functions

**b540** General metabolic functions

**b710** Mobility of joints functions

**b730** Muscle power functions

**b735** Muscle tone functions

**b760** Control of voluntary movements functions

**b765** Involuntary movements function

**b810** Protective functions of the skin

Other body functions

**Activity and participation**

**d110** Watching

**d115** Listening

**d120** Other purposeful sensing (mouthing, touching)

**d131** Learning through play

**d130** Copying

**d155** Acquiring skills

**d175** Solving problems

**d177** Making decisions

**d210** Undertaking a single task

**d220** Undertaking multiple tasks

**d230** Carrying out daily routine

**d235** Managing one’s behaviour

**d240** Handling stress and other psychological demands

**d310** Communicating with -- receiving -- spoken messages

**d315** Communicating with -- receiving -- non-verbal messages

**d330** Speaking

**d331** Preverbal vocalisation

**d335** Producing non-verbal messages

**d410** Changing basic body position

**d415** Maintaining a body position

**d420** Transferring oneself

**d430** Lifting and carrying objects

**d440** Fine hand use (picking up, grasping)

**d445** Hand and arm use

**d510** Washing oneself (bathing, drying, washing hands etc

**d520** Caring for body parts (brushing teeth, shaving, grooming etc)

**d550** Eating

**d560** Drinking

**d570** Looking after one’s health

**d620** Acquisition of goods and services (shopping etc)

**d630** Preparation of meals (cooking etc)

**d710** Basic interpersonal interactions

**d720** Complex interpersonal interactions

**d730** Relating with strangers

**d740** Formal relationships

**d820** School education

**d9** Community life

Other activities and participation

**Environmental Factors**

**e110** products for personal consumption (food, medicine)

**e1100** food

**e1101** drugs

**e115** for personal use in daily living

**e125** for communication

**e130** for education

**e310** support and relationships - immediate family

**e320** support and relationships - friends

**e330** support and relationships - people in position of authority

**e340** support and relationships - personal care providers and personal assistants

**e355** support and relationships - health professionals

**e360** support and relationships - other service professionals

**e410** Individual attitudes of immediate family members

**e420** Individual attitudes of friends

**e440** Individual attitudes of personal care providers and personal assistants

**e450** Individual attitudes of health professionals

**e455** Individual attitudes of health related professionals

**e460** Societal attitudes

**e465** Social norms, practices and ideologies

**e540** Transportation services, systems and policies

**e570** Social security services, systems and policies

**e575** General social support services, systems and policies

**e580** Health services, systems and policies

**e585** General education and training services, systems and policies

**e586** Special education and training services, systems and policies

Other environmental factors
